# Supplementary material for: Pharmacokinetic/Pharmacodynamic Analysis of Oral Calcium Fosfomycin: Are Urine Levels Sufficient to Ensure Efficacy for Urinary Tract Infections?
Source: Pharmaceutics. 2023 Apr 7;15(4):1185. doi: 10.3390/pharmaceutics15041185 (PMC10144240; doi:10.3390/pharmaceutics15041185)
Supplement: Supplementary file 1 [file pharmaceutics-15-01185-s001.zip › pharmaceutics-2251369-supplementary.pdf]

Supplementary material

Table S1. Stability data of fosfomycin in the urine samples.

**Condition**	**Result**
Freeze-thaw stability in matrix (30-4000 mg/L)	3 cycles, freezing at -80±10ºC, thawing at room temperature, polypropylene tubes
Short-term stability in matrix (30-4000 mg/L)	24 hours, room temperature polypropylene tubes
Processed samples stability (30-4000 mg/L)	136 hours at +10 ºC
Long-term stability in matrix at -25± 5ºC (30-4000 mg/L)	6 days in polypropylene tubes
Long-term stability in matrix at -80±10 ºC (30-4000 mg/L)	133 days in polypropylene tubes
